# CD154 blockade modulates the ratio of Treg to Th1 cells and prolongs the survival of allogeneic corneal grafts in mice

**DOI:** 10.3892/etm.2014.1527

**Published:** 2014-02-07

**Authors:** XIAOBO TAN, HUI ZENG, YING JIE, YINGNAN ZHANG, QING XU, ZHIQIANG PAN

**Affiliations:** 1 Beijing Ophthalmology and Visual Science Key Laboratory, Beijing Tongren Eye Center, Beijing Tongren Hospital, Capital Medical University, Beijing 100730, P.R. China; 2 Department of Ophthalmology, the Affiliated Hospital of Chengde Medical College, Chengde, Hebei 067000, P.R. China; 3 Institute of Infectious Diseases, Beijing Ditan Hospital, Capital Medical University, Beijing 100730, P.R. China

**Keywords:** CD154, corneal transplantation, mouse, regulatory T cells, T helper 1 cells

## Abstract

Administration of anti-CD154 monoclonal antibody (mAb) may prolong the survival of an allograft; however, the associated therapeutic mechanisms remain poorly understood. This study aimed to evaluate the effects of anti-CD154 mAb on T-cell responses in a mouse model of corneal allograft transplantation. BALB/c mice were transplanted with corneal grafts from C57BL/6 mice and treated intraperitoneally with 250 μg anti-CD154 mAb or isotype IgG on days 0, 3 and 6 post surgery. The transparency of the corneal grafts was evaluated for potential rejection signs by slit-lamp biomicroscopy and histopathology. The percentages of CD4^+^ T, Tim-3^+^CD4^+^ T helper (Th) 1 and CD4^+^CD25^+^Foxp3^+^ regulatory T cells (Tregs) in the spleen, ipsilateral draining lymph nodes and corneal grafts, and the frequency of splenic IFN-γ^+^ and IL-10^+^ expression in CD4^+^ T cells were determined by flow cytometry. Moreover, the ratio of Tregs to Th1 cells was calculated and the suppressive activity of splenic Tregs was measured. Anti-CD154 neutralization significantly prolonged the survival of the corneal allograft (P=0.0012) and reduced the numbers of inflammatory infiltrates in the corneal graft. In the spleen and lymph nodes, anti-CD154 treatment reduced the frequency of CD4^+^ T cells, Tregs and particularly Th1 cells. In the corneal allografts, anti-CD154 treatment downregulated graft-infiltrated CD4^+^ T cells and Th1 cells, but increased graft-infiltrated Tregs. Furthermore, anti-CD154 treatment increased the frequency of splenic IL-10^+^CD4^+^ T cells and decreased the concentration of splenic IFN-γ^+^CD4^+^ T cells. As a result, the ratio of Tregs to Th1 cells in the anti-CD154-treated recipients increased. Anti-CD154 treatment did not enhance the suppressive activity of Tregs in the recipients. The results indicate that the therapeutic effects of anti-CD154 mAb on prolonging the survival of the corneal allograft may be associated with an increased ratio of Tregs to Th1 cells in mice.

## Introduction

Immune rejection-mediated failures of corneal transplantation frequently occur (1). Although therapeutic strategies, including treatment with corticosteroids or other agents have improved the survival of grafts, patients with corneal transplantation may have problems, such as chronic graft loss and drug-associated adverse effects (2–4). Therefore, the development of novel therapies and the understanding of their therapeutic mechanisms are of great significance.

Activated T cells are known to be critical in allograft rejection. T-cell activation depends on the transfer of antigenic determinants presented by antigen-presenting cells (APCs) and costimulation signals that involve the interactions between costimulatory molecules on T cells and APCs, such as B7/CD28, CD40/CD154 and B7RP1/ICOS. Both are indispensable in the production of an effective immune response. Hence, therapeutic modulation of costimulation signals may control T-cell activation and allograft rejection (5,6). During the past 20 years, several therapeutic antibodies against costimulation signaling molecules have been developed and demonstrated to be effective in inhibiting allograft rejection, including corneal allograft rejection (7,8).

CD154 (CD40 ligand), mainly expressed on the surface of activated CD4^+^ T cells, is an attractive therapeutic target (9). Treatment with an anti-CD154 mAb to block CD40 and CD154 interaction alone or combined with additional approaches has been shown to be effective in preventing experimental allograft rejection (10–13). Administration of anti-CD154 mAb is also applied in the control of corneal allograft rejection (14–16). However, the effects of anti-CD154 mAb on allograft survival are unsatisfactory and the mechanism by which anti-CD154 mAb exerts its function in preventing allograft rejection remains unclear. Therefore, investigation of the underlying mechanisms of anti-CD154 mAb may be of benefit.

Previous studies have suggested that the therapeutic effects of anti-CD154 may be associated with the induction of T-cell anergy, the deletion of alloreactive CD4^+^ T cells, reductions in the levels of Th1 cytokine production or the suppression of ocular chemokine gene expression (17–19). Moreover, treatment with anti-CD154 mAb has been shown to enhance Treg response in a mouse model of islet allograft transplantation (20). Given that the rejection of normal-risk corneal grafts is usually slow, the determination of how anti-CD154 treatment affects the infiltration of alloreactive effector T cells and Tregs into the grafts is challenging.

In the present study, a high-responsive mouse model of corneal graft transplantation was employed to determine the effects of anti-CD154 treatment on Th1 and Tregs response. Our findings may provide novel insights into the mechanisms by which anti-CD154 modulates the survival of corneal allografts.

## Materials and methods

### Animals

Male wild-type C57BL/6 (H-2^b^) and BALB/c (H-2^d^) mice (age, 8–10 weeks) were obtained from the Experimental Animal Center of Capital Medical University (Beijing, China). The animals were housed in a specific pathogen-free facility. Mice were housed at 24°C under 12 h light/dark cycles with free access to food and water. The experimental procedures were ethically approved by the Animal Care and Research Committee of Capital Medical University (Beijing, China).

### Corneal transplantation

Recipient BALB/c mice were subjected to orthotopic penetrating transplantation of a corneal allograft from donor C57BL/6 mice or syngeneic grafts from BALB/c mice. Surgery was performed in the right eyes of individual mice, as described previously (21). Briefly, the donor corneas and the recipient graft bed were prepared by excising a 2×2-mm site in the central cornea. The donor button was then placed onto the recipient bed and secured with eight interrupted 11-0 nylon sutures. Following transplantation, the eyelids were closed for 3 days with tarsorrhaphy using 8-0 nylon sutures. The corneal sutures were not removed during the 4-week observation period.

### Experimental design and medical interventions

Two sets of corneal transplants were designed respectively. One set was used for clinical assessment of the grafts and the other set was used for the immunological tests. In each set, the allograft recipients were randomly injected intraperitoneally with 250 μg monoclonal anti-CD154 antibody (clone MR1) or control isotype hamster IgG antibody (both from Bio X Cell, West Lebanon, NH, USA) on days 0 (immediately after grafting), 3 and 6 following transplantation. The dose and time of medical intervention protocol used in this study were described previously (14,22). The syngeneic graft recipients did not receive anti-CD154 treatment. BALB/c mice transplanted with corneal allografts were randomly treated with anti-CD154 mAb or isotype IgG (n=10 per group). BALC/c mice that received syngeneic corneal grafts were left untreated as controls (n=10).

### Evaluation of corneal grafts

Corneal grafts were examined from day 3 post surgery. The degrees of graft opacity were scored under a slit-lamp biomicroscope (Topcon SL-1E; Topcon, Tokyo, Japan) twice per week for up to 4 weeks following transplantation in a blinded manner. The degrees of opacity were scored as follows: 0, clear; 1+, minimal superficial opacity; 2+, mild stromal opacity with pupil margin and iris vessels visible; 3+, moderate stromal opacity with only pupil margin visible, but iris vessels obscured; 4+, complete opacity with pupil and iris totally obscured. The onset of graft rejection was diagnosed as the time when the corneal opacity score increased to 3+ in a graft that was previously clear following transplantation, as previously described (23,24).

### Histopathology

Eyeballs were removed from individual recipients on day 14 post surgery and fixed in 10% buffered formalin. The paraffin-embedded tissue sections (5 μm) were stained with hematoxylin and eosin, and examined under a light microscope (Olympus BX51; Olympus, Tokyo, Japan).

### Flow cytometric analysis

Ipsilateral draining submandibular lymph nodes, cervical lymph nodes, spleens and corneal grafts were harvested from the mice before surgery and at weekly intervals up to 4 weeks after surgery. Splenic and lymph node single-cell suspensions were prepared. Spleens and lymph nodes were harvested under sterile condition and passed through a 200 mesh sieve to remove the tissue fragments. The red cells were lysed in 5 ml of Tris-ammonium chloride buffer (0.83% NH_4_Cl, 5 mM Tris buffer, pH 7.2) at 37°C for 5 min. Then lymphocytes were washed twice at 500 × g for 5 min and re-suspended in PBS at a concentration of 1×10^7^ cells/ml. Th1 cells were identified by surface staining with PerCP-anti-CD3, FITC-anti-CD4 and PE-anti-Tim-3. Tregs were surface stained first with APC-anti-CD4 and PerCP-anti-CD25 (eBioscience, Inc., San Diego, CA, USA) and then, following fixation and permeabilization, were intracellularly stained with Alexa Fluor 488-conjugated anti-Foxp3 (Biolegend, San Diego, CA, USA).

For intracellular cytokine staining, splenic single cells (1×10^6^/well) were stimulated in triplicate with Cell Stimulation Cocktail (eBioscience, Inc.) in 10% fetal bovine serum and RPMI-1640 medium (Gibco-BRL, Carlsbad, CA, USA) for 6 h. The cells were surface-stained with APC-anti-CD4, fixed, permeabilized, and then intracytoplasmically stained with FITC-anti-IFN-γ and PerCP-anti-IL-10.

Corneal single cells were prepared as previously described (25). Briefly, corneal tissues were dissected and cut into small sections. The corneal tissues were digested with 2 mg/ml collagenase type IV (Sigma-Aldrich, St. Louis, MO, USA) at 37°C for 1 h. Subsequently, the digested tissues were triturated through a 21-gauge needle and passed through a 70 μm cell filter (BD Falcon; Becton Dickinson, Franklin Lakes, NJ, USA). The isolated cells (5×10^4^) were surface stained with PerCP-anti-CD3, FITC-anti-CD4 and PE-anti-Tim-3, fixed, permeabilized and then intracellularly stained with Alexa Fluor 488-conjugated anti-Foxp3 (Biolegend).

All intracellular stains were performed using the FixPerm kit (The Foxp3/Transcription Factor Fixation/Permeabilization Concentrate and Diluent solutions; eBioscience, Inc.). Unless otherwise specified, all anti-mouse antibodies were obtained from eBiosciences. Proper isotype controls were used in each set of experiments. Samples were acquired on a FACS Calibur (BD Biosciences) and analyzed using Flowjo software (Tree-Star, Inc., Ashland, OR, USA).

### Suppression assay

For the suppression assay, CD4^+^CD25^+^ Tregs were isolated from the spleens of the allograft recipients that had been treated with anti-CD154 or isotype IgG 14 days following transplantation. CD4^+^CD25^−^ T cells were isolated from the spleens of naïve BALB/c mice. The isolation was conducted using a CD4^+^CD25^+^ Regulatory T cell Isolation kit, according to the manufacturer’s instruction (Miltenyi Biotec, Bergisch Gladbach, Germany). The purity of the prepared cells was >95%, as determined by flow cytometry. Fresh isolated CD4^+^CD25^−^ T cells were labeled with 0.5 μM carboxyfluorescein diacetate succinimidyl ester (Invitrogen Life Technologies, Carlsbad, CA, USA) and served as T effector (Teff) cells. After washing, 1×10^5^ Teff cells were co-cultured in triplicate with 2×10^5^ mitomycin-treated C57 BL/6 splenocytes in the presence or absence of Tregs for 72 h. The ratio of Tregs to Teff cells varied from 1:1 to 1:8. The alloantigen-stimulated Teff cell proliferation was determined by flow cytometry and suppression was calculated using the following formula: Suppression (%) = (Teff proliferation without Tregs - Teff proliferation with Tregs)/(Teff proliferation without Tregs) × 100.

### Statistical analysis

Data are presented as the mean ± standard deviation. The survival curves of corneal grafts in different groups of mice were established by Kaplan-Meier analysis, and the difference among the different groups of mice was determined by the log-rank test. The difference in the frequency of T cells among different groups of mice was determined by analysis of variance with Bonferroni corrections or Student’s t-test. All analyses were performed using GraphPad Prism 5.0 software (GraphPad Software, Inc., La Jolla, CA, USA). P<0.05 was considered to indicate a statistically significant difference.

## Results

### Anti-CD154 neutralization prolongs survival of the corneal allograft and prevents corneal inflammation in mice

**Figure 1:**
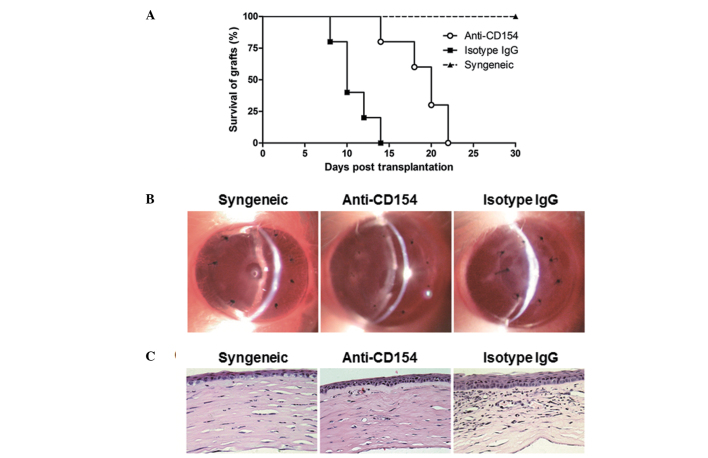
Analysis of corneal survival and transparency following transplantation. BALB/c mice transplanted with corneal allografts were randomly treated with anti-CD154 mAb or isotype IgG (n=10 per group). BALC/c mice that received syngeneic corneal grafts were left untreated as controls (n=10). The survival of the corneal grafts was monitored for up to 4 weeks post surgery. (A) Median survival time of the grafts. Kaplan-Meier curves were plotted to evaluate the graft survival. Log-rank test was performed to evaluate statistical significance (P=0.0012). (B) Representative slit-lamp biomicroscopy results of the grafts on day 14 following transplantation (magnification, ×10). (C) Histopathology results of the grafts (n=5 per group) on day 14 post surgery (magnification, ×1000).

The effects of short term treatment with anti-CD154 mAb on corneal allograft rejection were assessed. The survival time of the transplanted corneal in each group was measured at various time points following transplantation. It was observed that the syngeneic grafts in the recipients without any special treatment remained transparent throughout the 4-week observation period. All the corneal allografts were rejected eventually, but the median survival time of the grafts in anti-CD154 neutralization mice was longer than that of the isotype IgG-treated mice (20 days vs. 10 days, P=0.0012; Fig. 1A). Corneal grafts in the mice treated with isotype IgG began to lose their transparency at day 8 following surgery and survived for 14 days at most. By contrast, the onset of corneal allograft rejection in the anti-CD154-treated mice was delayed to day 14 post surgery (1 week following treatment withdrawal) and survived for >3 weeks post surgery. Furthermore, the transparency of corneal grafts was evaluated by slit-lamp biomicroscopy on day 14 following transplantation. As shown in Fig. 1B, the corneal allografts in the anti-CD154-treated mice appeared to have greater clarity than those of the isotype IgG-treated mice. In addition, the transparency of the corneal grafts was further analyzed by histopathology. As shown in Fig. 1C, the corneal allografts in the anti-CD154-treated mice remained intact with a few inflammatory infiltrations and neovessels in their stromas, which were similar to those of the syngeneic grafts. By contrast, the allografts in the isotype IgG-treated mice displayed edematous corneal stromas with a large quantity of inflammatory cells and neovascularizations. Given the significant difference between anti-CD154-treated and isotype IgG-treated mice on day 14 following transplantation, this time-point was selected for the following experiments.

### Anti-CD154 neutralization downregulates the frequency of peripheral Tregs following corneal transplantation

**Figure 2:**
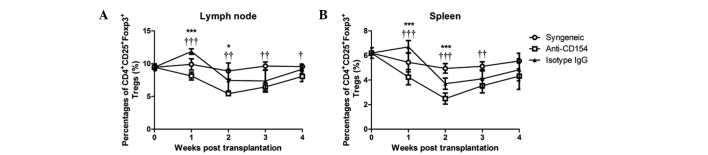
Percentage of peripheral Tregs during the observation period following transplantation. Ipsilateral lymph nodes and spleens were obtained from naïve BALB/C mice, syngeneic corneal graft recipient mice and allograft recipient mice treated with anti-CD154 or isotype IgG at weekly intervals (n=5 per group). Subsequently, the frequency of CD25^+^Foxp3^+^ Tregs in CD4^+^ cells was determined by flow cytometry. Quantitative flow cytometry results are shown. Data are expressed as the mean ± standard deviation of three independent experiments. ^*,†^P<0.01; ^**,††^P<0.001; ^***,†††^P<0.0001; ^*^anti-CD154 group vs. isotype IgG group; and ^†^anti-CD154 group vs. syngeneic group.

To investigate the role of Tregs in anti-CD154 mAb-mediated delayed rejection of the corneal allograft, the frequency of Tregs in the spleens and lymph nodes were analyzed by flow cytometry. Quantitative results are shown in Fig. 2. The concentrations of CD4^+^CD25^+^Foxp3^+^ Tregs in the spleens and lymph nodes in the syngeneic graft mice showed no difference at any time-points. Following allogeneic corneal transplantation, the concentration of Tregs marginally increased 1 week following surgery and decreased 2 weeks post surgery in the isotype IgG-treated mice, followed by a gradual return to a concentration similar to that prior to transplantation. By contrast, the percentage of Tregs in the anti-CD154-treated mice gradually deceased in the first 2 weeks and then increased to a concentration similar to that prior to surgery. Statistically, the percentage of Tregs in the spleens and lymph nodes in anti-CD154-treated mice were significantly lower than those in the isotype IgG-treated mice at 1 and 2 weeks post surgery (P<0.0001 at week 1, P<0.01 at week 2), and significantly lower than those in syngeneic graft mice from 1 to 4 weeks post surgery (P<0.0001 at week 1, P<0.001 at week 2 and week 3, P<0.01 at week 4). Therefore, anti-CD154 neutralization downregulated the peripheral frequency of Tregs in mice following corneal transplantation.

### Regulatory effects of anti-CD154 on the CD4^+^ T cells, Tregs and Th1 cells in mice following corneal transplantation

**Figure 3:**
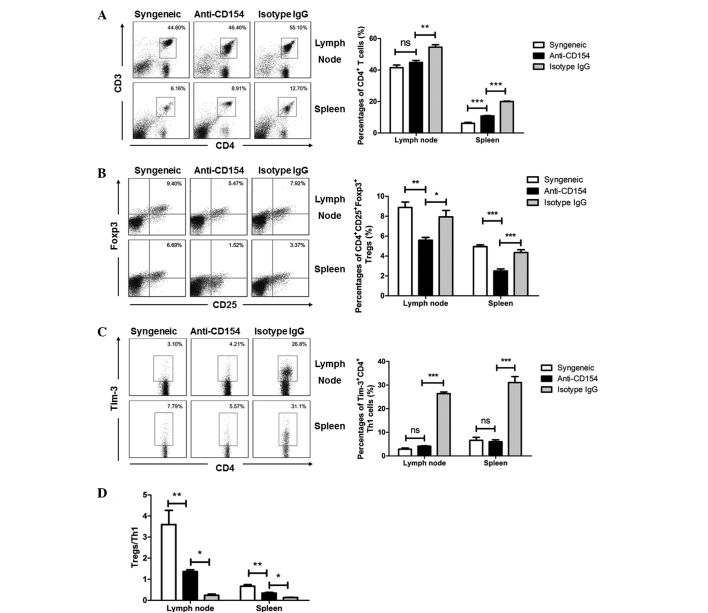
Flow cytometric analysis of the concentrations of CD4^+^ T, CD4^+^CD25^+^Foxp3^+^ Tregs and Tim-3^+^ CD4^+^ Th1 cells in the spleens and lymph nodes of mice. On day 14 following transplantation, mice were sacrificed and the ipsilateral lymph nodes and spleens were obtained from syngeneic corneal graft recipient mice and allograft recipient mice treated with anti-CD154 or isotype IgG (n=5 per group). The frequencies of CD4^+^ T, CD4^+^CD25^+^Foxp3^+^ Tregs and Tim-3^+^ CD4^+^ Th1 cells were determined by flow cytometry. (A) Representative and quantitative flow cytometry results for CD4^+^ T cells in the spleens and lymph nodes. Numbers within each quadrant represent the percentages of CD4^+^ T cells. (B) Representative and quantitative flow cytometry results for CD4^+^CD25^+^Foxp3^+^ Tregs in the spleens and lymph nodes. Numbers within each quadrant represent the percentages of CD4^+^CD25^+^Foxp3^+^ Tregs. (C) Representative and quantitative flow cytometry results for Tim-3^+^ CD4^+^ Th1 cells in the spleens and lymph nodes. Numbers within each quadrant represent the percentages of Tim-3^+^ CD4^+^ Th1 cells. (D) Ratio of Tregs to Th1 cells in spleens and lymph nodes. Data are presented as the mean ± standard deviation. Analysis of variance with Bonferroni corrections was performed to evaluate statistical significance. ^*^P<0.01; ^**^P<0.001; ^***^P<0.0001. NS, no significance.

**Figure 4:**
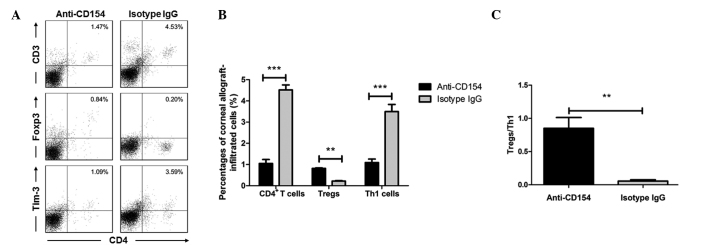
Flow cytometric analysis of corneal allograft-infiltrated CD4^+^ T, CD4^+^CD25^+^Foxp3^+^ Tregs and Tim-3^+^ CD4^+^ Th1 cells in mice. Corneal grafts were harvested from recipient mice treated with anti-CD154 or isotype IgG 14 days post surgery (n=5 per group). The frequencies of CD4^+^ T, CD4^+^CD25^+^Foxp3^+^ Tregs and Tim-3^+^ CD4^+^ Th1 cells were analyzed by flow cytometry. (A) Representative flow cytometry results. Numbers within each quadrant represent the percentages of CD4^+^ T, CD4^+^CD25^+^Foxp3^+^ Tregs and Tim-3^+^ CD4^+^ Th1 cells. (B) Quantitative results of the frequencies of CD4^+^ T, Th1 cells and Tregs in corneal grafts. (C) Ratio of Tregs to Th1 cells in corneal grafts. Data are presented as the mean ± standard deviation. P-values were calculated using Student’s t-test. ^**^P<0.001; ^***^P<0.0001.

The percentages of CD4^+^ T cells, Tregs and Th1 cells in the spleens, lymph nodes and corneal grafts following transplantation were analyzed by flow cytometry. Fig. 3 shows the flow cytometry results in the spleens and lymph nodes, and Fig. 4 shows the flow cytometry results in the corneal grafts (data of the syngeneic graft group were not shown due to lack of cells). It was identified that anti-CD154 treatment decreased the systemic total CD4^+^ T-cell frequency. The percentages of CD4^+^ T cells in the anti-CD154-treated mice were similar to those in the syngeneic graft mice, and significantly lower than those in the isotype IgG-treated mice (Fig. 3A, P<0.001 in lymph node and P<0.0001 in spleen; Figs. 4A and B, P<0.0001). Th1 cells have been considered as critical for allograft rejection; therefore, the correlations between Th1 cells and Tregs in the spleens, lymph nodes and corneal grafts of different groups of mice were determined. Following anti-CD154 neutralization, the percentages of lymph nodes and splenic Tregs were significantly lower than those in the syngeneic graft and the isotype IgG-treated allogeneic graft groups of mice (Fig. 3B). However, the concentration of Tregs in the corneal grafts from the anti-CD154-treated mice was upregulated (Fig. 4A and B). The percentages of lymph node and splenic Th1 cells following neutralization were similar to those in the syngeneic graft group of mice, but significantly lower than those in the isotype IgG-treated allogeneic graft group of mice in all three tissues (Figs. 3C, 4A and 4B; P<0.0001). Furthermore, the ratios of Tregs to Th1 cells in the lymph nodes, spleens and corneal grafts from the anti-CD154-treated mice were significantly lower than those of the syngeneic graft mice, but significantly greater than those of the isotype IgG-treated mice (Fig. 3D, anti-CD154 group vs. syngeneic group, P<0.001; anti-CD154 group vs. isotype IgG group, P<0.01. Fig. 4C, P<0.001). These results indicate that a low Treg:Th1 ratio may have contributed to the inhibition of corneal allograft rejection in mice.

### Anti-CD154 neutralization modulates Treg- and Th1-associated cytokines in mice following corneal transplantation

**Figure 5:**
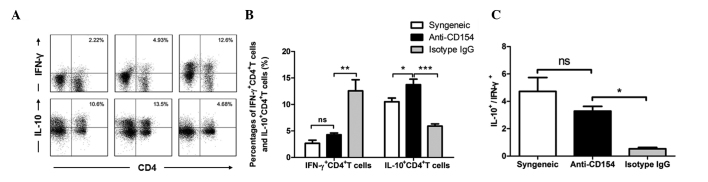
Analysis of splenic IFN-γ- and IL-10-expressing CD4^+^ T cells in mice. On day 14 following transplantation, splenocytes were isolated from each group of mice (n=5 per group) and stimulated in triplicate with a cell stimulation cocktail for 6 h. Subsequently, the frequencies of splenic IFN-γ- and IL-10-expressing CD4^+^ T cells were determined by flow cytometry. (A) Representative results of flow cytometric analysis. Numbers within each quadrant represent the percentages of IFN-γ- and IL-10-expressing CD4^+^ T cells, respectively. (B) Quantitative results of the frequency of IFN-γ- and IL-10-expressing CD4^+^ T cells. (C) Ratio of IL-10^+^ to IFN-γ^+^ CD4^+^ T cells in mice with allogeneic corneal transplants. Analysis of variance with Bonferroni corrections was performed to evaluate statistical significance. ^*^P<0.01; ^**^P<0.001; ^***^P<0.0001; NS, no significance.

The frequencies of IFN-γ- and IL-10-expressing CD4^+^ T cells in the spleens from different groups of mice were determined by flow cytometry. Representative and quantitative flow cytometry results are shown in Fig 5. The percentage of splenic IFN-γ^+^CD4^+^ T cells in the anti-CD154-treated mice was not significantly different from that in the syngeneic graft group of mice, but significantly lower than that in the isotype IgG-treated group of mice (Fig. 5A and B; P<0.001). By contrast, the concentration of IL-10^+^CD4^+^ T cells in mice following anti-CD154 neutralization was significantly higher than those in the other two groups of mice (anti-CD154 group vs. syngeneic group, P<0.01; anti-CD154 group vs. isotype IgG group, P<0.0001). As shown in Fig. 5C, the ratio of IL-10^+^CD4^+^ T cells to IFN-γ^+^CD4^+^ T cells in anti-CD154-treated mice was significantly greater than that in the isotype IgG-treated mice (P<0.01) In addition, there was no significant difference in this ratio between the anti-CD154-treated and syngeneically grafted mice. Therefore, anti-CD154 treatment significantly increased the expression levels of IL-10^+^ by CD4^+^ T cells, whereas it downregulated the allograft-mediated expression of IFN-γ^+^ by CD4^+^ T cells. Anti-CD154 treatment increased the ratio of IL-10^+^CD4^+^ T cells to IFN-γ^+^CD4^+^ T cells in mice with allogeneic corneal transplants.

### Anti-CD154 neutralization does not enhance the suppressive activity of Tregs

**Figure 6:**
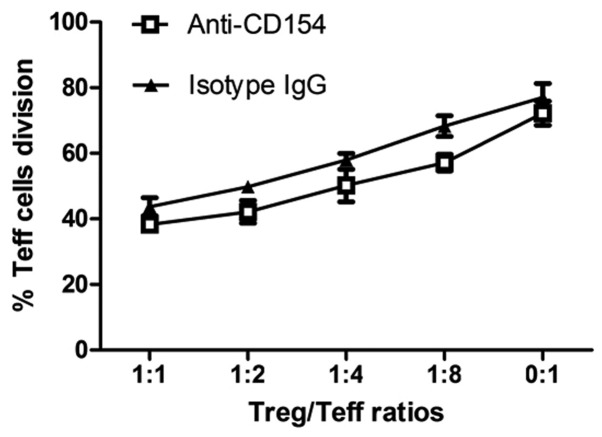
Treg suppressive activity analysis. CD4^+^CD25^+^ Tregs were isolated from allograft recipients that had been treated with anti-CD154 mAb or isotype IgG on day 14 post surgery. CD4^+^CD25^−^ T effector cells were isolated from naïve BALB/c mice, labeled with 0.5 μM CFSE, and then stimulated with mitomycin C-treated C57BL/6 splenocytes for 72 h in the presence or absence of Tregs at the indicated ratio. The proliferation of CFSE-labeled CD4^+^CD25^−^ T effector cells was measured by flow cytometry. Quantitative results are shown. Data presented are the mean ± standard deviation of three independent experiments. Student’s t-test was performed to evaluate statistical significance. ^*^P<0.01; ^**^P<0.001; ^***^P<0.0001. CFSE, carboxyfluorescein diacetate succinimidyl ester.

Since Tregs are able to suppress the proliferation of effector T cells, the effects of anti-CD154 neutralization on the suppressive activity of Tregs were further examined. Although the presence of Tregs in the isotype IgG-treated mice appeared to have marginally increased the proliferation of Teff cells compared with that for the Tregs from the anti-CD154-treated mice, no statistically significant difference was identified in the suppressive activity of Tregs from the two groups of mice (Fig. 6). These results indicate that treatment with anti-CD154 mAb does not enhance the suppressive activity of Tregs in mice with allogeneic corneal transplantation.

## Discussion

Antibody-based immunotherapies have been widely used for protecting organ transplants from immune rejection. However, there are only a small number of studies concerning the application of antibodies in corneal transplantation (26,27). In the present study, it was demonstrated that treatment with anti-CD154 mAb (three times) following allogeneic corneal transplantation prolonged the survival of corneal allografts in mice, and the survival rate at 2 weeks following transplantation was 80%. These findings suggest that therapy with anti-CD154 mAb may not have induced permanent transplant tolerance but rather prolonged immune suppression. Anti-CD154 treatment inhibited T-cell activation and prolonged the survival of allografts in the recipients (28). Hence, the modest prolonged effects of graft survival were expected.

In the present study, the immunosuppressive effects of anti-CD154 mAb were confirmed by the findings of CD4^+^ T cells and their subsets following corneal transplantation. CD4^+^ T cells are important in controlling the different mechanisms involved in corneal graft rejection and are thus a target for evaluating therapeutic interventions designed to prevent corneal allograft rejection (29). In the present study, it was observed that treatment with anti-CD154 mAb significantly reduced the frequency of total CD4^+^ T cells that infiltrated the ipsilateral draining lymph nodes, spleens and corneal grafts. Engagement of T-cell receptor on naïve CD4^+^ T cells by antigen determinant presented by major histocompatibility complex molecules, may have activated the differentiation of T cells into different subsets. Th1 cells are the sole mediators of delayed type hypersensitivity response, which has been identified to be closely associated with corneal allograft rejection (30). In the present study, treatment with anti-CD154 mAb significantly reduced the frequency of splenic, lymph node and corneal graft-infiltrating Th1 cells in mice. In parallel, the concentration of splenic IFNγ^+^CD4^+^ T cells was significantly reduced. These results support the theory that Th1 cells are the primary, if not sole T-cell population required for corneal allograft rejection, and the inhibition of Th1 responses by CD154 neutralization is associated with long-term survival of the corneal allograft.

The role of Tregs in establishing and maintaining immune tolerance is increasingly appreciated (31–33). Previous studies in mice have shown that treatment with anti-CD154 mAb enhanced the Treg response and contributed to its inhibitory effect on allograft rejection (34,35). Notably, anti-CD154 mAb treatment reduced the concentration of Tregs in the spleen and lymph nodes at the early stage of corneal allograft rejection in the present. These results are consistent with findings in CD154-knockout mice (36). Furthermore, the kinetics of peripheral Tregs during the entire observation period were investigated. The levels of Tregs were observed to be reduced following CD154 blockade and restored at the end of treatment. Moreover, the frequency of splenic IL-10^+^CD4^+^ T cells was increased at the early stage of corneal allograft rejection. Given that IL-10 may be produced by Tregs and other regulatory T cells, such as Th2 and Tr1, and that IL-10 is a potent anti-inflammatory cytokine, the increased concentration of IL-10^+^CD4^+^ T cells may have contributed to the inhibition of corneal allograft rejection. Notably, no significant differences in the suppressive activity of Tregs from the anti-CD154-treated and isotype IgG-treated control mice were observed. These results re-confirm the immunosuppressive effects of anti-CD154 mAb on corneal allograft rejection and indicate that the CD40/CD40L pathway may have marginal regulatory effects on Treg suppressive activity.

However, the effects of Tregs should not be excluded as the balance between pro-inflammatory Th1 cells and anti-inflammatory Tregs may have determined the survival of the allograft. Accordingly, in the present study was found that the inhibitory effects of anti-CD154 mAb treatment on the Th1 response were stronger than that on Tregs. As a result, the ratio of Tregs to Th1 cells in the anti-CD154-treated mice was higher than that in the isotype IgG-treated control mice. Therefore, anti-CD154 mAb treatment preferentially inhibited the pro-inflammatory Th1 responses and regulated the balance of anti-inflammatory Tregs and pro-inflammatory Th1 responses, resulting in the prolonged survival time of the corneal allografts in mice.

The cornea has been considered to be blood-free and possibly lymph vessel-free, and the corneal infiltrating cells are mainly derived from neovascularized vessels, which reflects the change in the peripheral cells (37). However, treatment with anti-CD154 mAb increased the frequency of Tregs in corneal grafts. It is possible that following the blockade of CD40 and CD40L interaction, Tregs redistribute in the recipients and tended to migrate into the corneal grafts. Therefore, these results suggest that CD40 and CD40L interaction not only regulates Treg differentiation, but also modulates Treg migration *in vivo*.

In conclusion, our data indicate that treatment with anti-CD154 mAbs inhibited acute corneal allograft rejection in mice by increasing the ratio of Tregs to Th1 cells in the peripheral lymphoid organs and the corneal graft. Our findings also suggest that modulation of the balance between anti-inflammatory Tregs and the inflammatory Th1 response may have determined the survival of the corneal allograft. Therefore, we speculate that the effects of anti-CD154 mAb on graft survival may be improved by alternatively adding an additional agent that may strengthen the Treg response. It should be addressed that, besides Tregs and Th1 cells, other subsets of T cells, such as Th2, Th9, Th17 and Th22 may be recruited to regulate allograft rejection. Therefore, further studies are required to investigate the role of anti-CD154 mAb on these factors in corneal allograft rejection.
